# The Development of a Complex FEP Program at Semmelweis University

**DOI:** 10.1192/j.eurpsy.2022.86

**Published:** 2022-09-01

**Authors:** V. Simon, L. Herman, E. Vass, G. Csukly, R.I. Zsigmond, J. Réthelyi

**Affiliations:** Semmelweis University, Department Of Psychiatry And Psychotherapy, Budapest, Hungary

**Keywords:** First Episode Psychosis, schizophrénia, therapy

## Abstract

Introduction First episode psychosis (FEP) programs target the first 3-5 years after the first manifestation of a psychotic episode. Although follow-up results after 10-15 years are mixed, reported results of the first 5-10 years are promising in improving relapse prevention, functional outcomes and reducing mental health care costs, compared to treatment as usual. Objectives Our objective was to develop the first complex FEP program in Hungary. Methods Male and female inpatients, (age:17-40years), hospitalized due to a first psychotic episode and consenting to participate, were included, since 2019 October. Drug induced psychosis and organic background in the etiology of the psychotic episode were excluded. Duration of the program is 2-3 years, based on the individual needs of the patients. The program provides detailed clinical and neuropsychological examinations, MRI, pharmacotherapy, various psychosocial interventions, and support group for relatives. Results Twenty-eight patients [15 male,13 female, mean age=22,4 (18-40)years] were included. The program has been repeatedly limited by COVID-19 pandemic. Psychiatric control, pharmacotherapy, psychoeducation and supportive therapy, however could be continously provided via telepsychiatry, even during the lockdown. During the first 2 years, 90% of the patients remained in treatment, showed good compliance with pharmacotherapy; 10% of the patients were rehospitalized due to relapse. In the presentation, besides clinical experiences, preliminary clinical outcome data will also be presented. Conclusions The first complex FEP program in Hungary, although limited repeatedly by the pandemic, show promising clinical results with low relapse rates and high level of adherence to therapy in the first 2 years after a first psychotic episode.

**Disclosure:**

No significant relationships.

